# Pregnancy and weaning regulate human maternal liver size and function

**DOI:** 10.1073/pnas.2107269118

**Published:** 2021-11-22

**Authors:** Alexandra Q. Bartlett, Kimberly K. Vesco, Jonathan Q. Purnell, Melanie Francisco, Erica Goddard, Xiangnan Guan, Andrea DeBarber, Michael C. Leo, Eric Baetscher, William Rooney, Willscott Naugler, Alexander R. Guimaraes, Patrick Catalano, Zheng Xia, Pepper Schedin

**Affiliations:** ^a^Department of Cell, Developmental, and Cancer Biology, Oregon Health & Science University, Portland, OR 97239;; ^b^Center for Health Research, Kaiser Permanente Northwest, Portland, OR 97227;; ^c^Knight Cardiovascular Institute, Oregon Health & Science University, Portland, OR 97239;; ^d^Public Health Sciences Division/Translational Research Program, Fred Hutchinson Cancer Research Center, Seattle, WA 98109;; ^e^Computational Biology Program, Oregon Health & Science University, Portland, OR 97201;; ^f^Department of Chemical Physiology and Biochemistry, Oregon Health & Science University, Portland, OR 97239;; ^g^Advanced Imaging Research Center, Oregon Health & Science University, Portland, OR 97239;; ^h^Department of Medicine, Division of Gastroenterology and Hepatology, Oregon Health & Science University, Portland, OR 97239;; ^i^Department of Diagnostic Radiology, Oregon Health & Science University, Portland, OR 97239;; ^j^Mother Infant Research Institute, Department of Obstetrics and Gynecology, Tufts University School of Medicine, Boston, MA 02111;; ^k^Department of Molecular Microbiology and Immunology, Oregon Health & Science University, Portland, OR 97273;; ^l^Knight Cancer Institute, Oregon Health & Science University, Portland, OR 97201;; ^m^Young Women’s Breast Cancer Translational Program, University of Colorado Anschutz Medical Campus, Aurora, CO 80045

**Keywords:** liver, pregnancy, bile acids, maternal health

## Abstract

During pregnancy, the rodent liver undergoes hepatocyte proliferation and increases in size, followed by weaning-induced involution via hepatocyte cell death and stromal remodeling, creating a prometastatic niche. These data suggest a mechanism for increased liver metastasis in breast cancer patients with recent childbirth. It is unknown whether the human liver changes in size and function during pregnancy and weaning. In this study, abdominal imaging was obtained in healthy women at early and late pregnancy and postwean. During pregnancy time points, glucose production and utilization and circulating bile acids were measured. Independently of weight gain, most women’s livers increased in size with pregnancy, then returned to baseline postwean. Putative roles for bile acids in liver growth and regression were observed. Together, the data support the hypothesis that the human liver is regulated by reproductive state with growth during pregnancy and volume loss postwean. These findings have implications for sex-specific liver diseases and for breast cancer outcomes.

## Significance

These human data are consistent with reproductive control of liver size and function in women and concur with recent observations in rodents, suggesting a conserved liver biology. The question of whether this described liver biology has implications for maternal health during pregnancy or sex-specific risk for liver disease remains to be determined. However, our evidence suggestive of weaning-induced liver involution in women may lead to improved understanding of the high rates of liver metastasis observed in young postpartum breast cancer patients.

Sex-specific differences in liver disease have been attributed to sexual dimorphisms in steroid production, metabolic enzymes, and behavior patterns (1). Whether a pregnancy cycle contributes to sex-specific liver disease remains largely unexplored; however, a previously unrecognized liver biology linked to reproductive status has been reported in rodents (2). This rodent study found that during pregnancy and lactation, hepatocytes proliferated and entered a higher anabolic state accompanied by an overall increase in liver size. Upon weaning, hepatocytes rapidly underwent programmed cell death, liver metabolism shifted toward catabolism, and the liver regressed to its prepregnant size in a process referred to as weaning-induced liver involution (2). In mice, liver involution promoted breast cancer outgrowth in the liver, suggesting a pathophysiological consequence of liver involution (2, 3).

Notably, young women diagnosed with breast cancer in the postpartum period were found to be at increased risk for liver metastasis (2). Taken together, these findings suggest that weaning-induced liver involution, which we predict would return the enlarged liver to its prepregnant, prelactational state, may create a prometastatic liver niche in women. However, it is unknown whether the human liver changes in size and function across a reproductive cycle, as expected if the liver is tuned to meet the unique metabolic demands of pregnancy, lactation, and weaning. Such evidence would corroborate findings in rodents and would be foundationally important for future studies of liver health in women.

To investigate the impact of reproductive state on liver size and function in women, we conducted a prospective study of healthy pregnant women using magnetic resonance and spectroscopy imaging of the liver and compared findings to a validated rodent model. Here, we show that the human female liver is regulated in both size and function by reproductive state and provide evidence of weaning-induced liver involution in humans. Furthermore, our data provide a hypothesis to explain the increased liver metastasis observed in postpartum breast cancer patients, as well as having potentially broader implications for the understanding of sex-specific liver diseases.

## Results

**Fig. 1.**
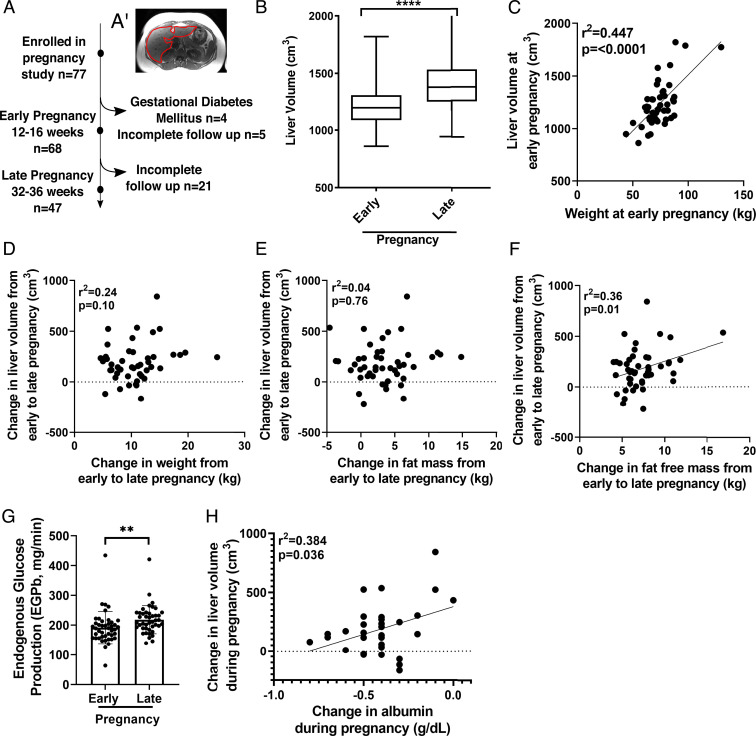
Liver changes during pregnancy. (*A*) Diagram of the observational study. (*A*’) Liver MRI cross-section with liver outlined in red. (*B*) Average liver volume at early and late pregnancy (*n* = 47; ****P < 0.0001 by two-tailed paired *t* test). (*C*) Pearson’s correlation of liver volume and BMI at early pregnancy (*n* = 47). Pearson’s correlation of change in liver volume with change in weight (*D*), fat mass (*E*), and fat-free mass (*F*) (*n* = 47). (*G*) EGP-b at early and late pregnancy (*n* = 47; **P < 0.01 by two-tailed paired *t* test). (*H*) Pearson’s correlation of change in liver volume and change in albumin (*n* = 30).

In total, 47 healthy pregnant women completed early (12 to 16 wk gestation) and late pregnancy (32 to 36 wk gestation) study visits (Fig. 1*A*). Study participants underwent liver MRI (Fig 1*A*), provided blood samples, had insulin sensitivity assessed via hyperinsulinemic-euglycemic clamp, and completed body composition analyses. Participant demographics are shown in *SI Appendix*, Table S1.

To assess whether liver size is increased during pregnancy, we measured livers via MRI and found that, on average, liver volumes increased 15% (182 cm^3^ ± 197 cm^3^) from early to late pregnancy (*P* < 0.0001) (Fig. 1*B*). Average liver size at early pregnancy was 1,239 cm^3^ ± 220.8 cm^3^ and at late pregnancy was 1,421 cm^3^ ± 298.6 cm^3^ (Fig. 1*B*).

Because liver size is attuned to overall body size via the “hepatostat” (4), we next determined whether the increase in liver size from early to late pregnancy correlated with increased body mass of pregnancy. First, we investigated the existence of the “hepatostat” at baseline, using body weight at the early pregnancy visit as a baseline surrogate, as pregnancy-related weight gain is minimal at this time point (5). Liver volume at early pregnancy correlated with body weight (Fig. 1*C*), confirming previous studies in nonpregnant individuals (4). In contrast, the change in liver volume during pregnancy did not correlate with gestational weight gain (Fig. 1*D*). Furthermore, we found no relationships between pregnancy liver volume change and change in total fat mass (Fig. 1*E*), subcutaneous abdominal, or visceral adipose tissue (VAT) volumes (Table 1). However, the change in a woman’s fat-free mass, which includes liver, fetal tissue, placenta, and plasma, did correlate with change in liver size (Fig. 1*F*). The association between change in fat-free mass and liver volume is confounded as fat-free mass is not an independent variable from liver mass. In sum, these data suggest that liver size increase during pregnancy is unlinked to overall body size; that is, it is not controlled by the “hepatostat” mechanism. Rather, these data may reflect an unrecognized, reproductive state–controlled program regulating liver size during pregnancy.

**Table 1.** Change in liver volume correlated with measures of body composition and metabolism

Variable	Mechanism of collection	Sample size	Pearson correlation coefficient	*P* value
Body composition				
Change in weight	Scale	47	0.260	0.078
Change in BMI	Scale, stadiometer	47	0.213	0.150
Change in fat mass	BODPOD	47	0.077	0.605
Change in fat-free mass	BODPOD	47	**0.335**	**0.021**
Change in SAT	MRI	47	0.123	0.409
Change in VAT	MRI	47	0.245	0.097
Change in IHL	H-MR spectroscopy	47	−0.035*	0.814*
Metabolism				
Change in M value	Hyperinsulinemic- euglycemic clamp			
		43	−0.015	0.926
Change in EGP	Hyperinsulinemic- euglycemic clamp			
		43	−0.047*	0.763*
Change in Rd	Hyperinsulinemic- euglycemic clamp			
		43	0.053	0.736
Change in fasting insulin	Blood draw	45	0.095	0.537
Change in total cholesterol	Blood draw	45	0.062	0.684
Change in triglycerides	Blood draw	45	0.176	0.248
Change in LDL	Blood draw	45	−0.119	0.438
Change in HDL	Blood draw	45	0.103	0.500
Change in very low density lipoprotein	Blood draw	45	−0.103	0.500

Bold text indicates that the change fat free mass was the only variable that reached statistical significance.

* These analyses were done with Spearmen Correlation.

We next asked if metabolic measures were associated with liver volume change and found no relationship with cholesterol concentrations or with measures of insulin sensitivity, that is, endogenous glucose production (EGP) and glucose disposal rate (Rd) (Table 1). We also found no relationship between change in liver volume and change in intrahepatic lipid (IHL) content (Table 1). Assessment of IHL content in rodents also showed no change in IHL during pregnancy (*SI Appendix*, Fig. S1). In sum, we observed that the increase in human liver volume with late pregnancy occurred independent of weight gain of pregnancy, various other measures of body composition, circulating metabolites, and IHL storage.

In rodents, hepatocyte proliferation contributes to increased liver size and metabolic output during pregnancy and lactation (2, 6). Obtaining timed liver biopsies would be the most direct way to investigate hepatocyte proliferation during pregnancy in women; however, liver biopsies were not performed in our study for participant safety. Thus, we indirectly assessed for increased hepatocyte number by evaluating hepatocyte function. We found evidence for increased liver output as measured by increases in EGP (Fig. 1*G*) and serum albumin concentration (Fig. 1*H*), two surrogates of liver function (7, 8). Of note, an additional contributor to increased liver volume during pregnancy is increased blood flow, which rises ∼50% by late pregnancy (9). However, increased blood flow during pregnancy is not reported to associate with elevated hepatocyte metabolic output. In sum, these data are consistent with an increase in liver size and synthetic capacity during pregnancy, which may be due to increased hepatocyte proliferation as observed in rodents. Additional studies are required to determine if hepatocyte proliferation is increased during pregnancy in women.

**Fig. 2.**
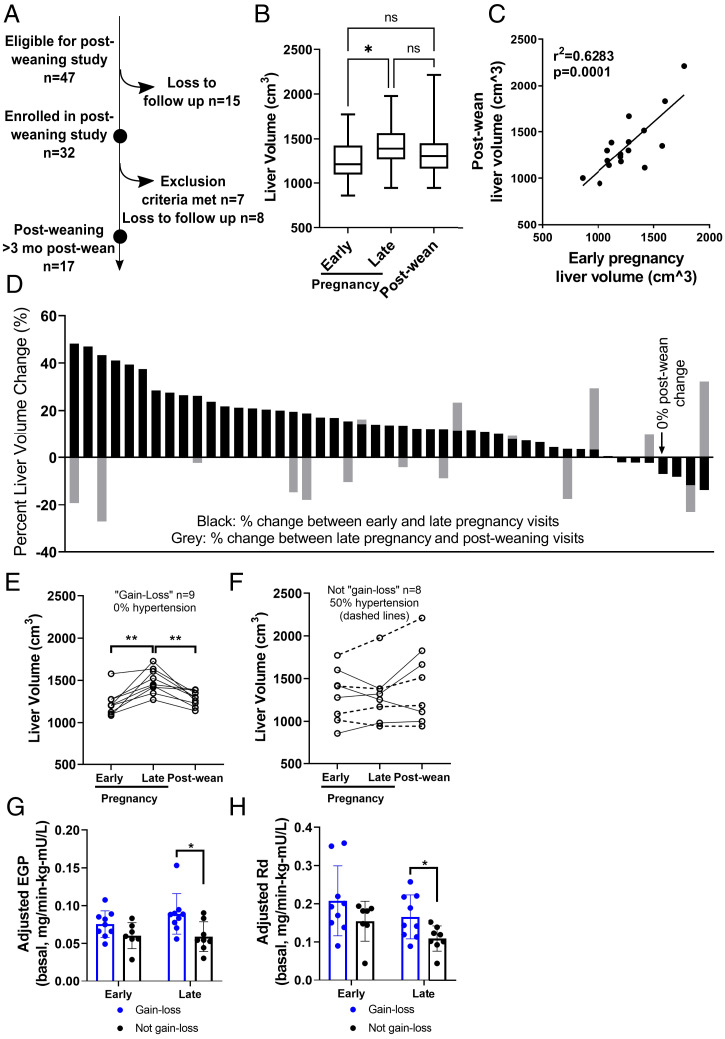
Human liver volumes postwean. (*A*) Diagram for postwean observational study. (*B*) Liver volume at early, late, and postwean time points (*n* = 17). (*C*) Pearson’s correlation of liver volumes at early pregnancy and postwean (*n* = 17). (*D*) Liver volume change between early and late pregnancy (black bars) and between late pregnancy and postwean (gray bars) per participant. Primary pattern (*E*) and secondary patterns (*F*) of liver volume change with pregnancy and postwean. Dashed lines show participants with hypertension (paired *t* test). EGP (*G*) and glucose disposal rate, Rd, (*H*) in women in gain–loss group compared to women not in gain–loss group. Pearson’s correlation. *P* value: * < 0.05, ** < 0.01.

We next looked for evidence of weaning-induced liver involution in women, a biology not previously described in humans. Of the 47 women who participated in our pregnancy study, 36% completed a liver MRI >3 mo postwean (median 5.7 mo) (Fig. 2*A*). Liver volumes trended toward a decrease in size between late pregnancy and postwean (Fig. 2*B*), and postwean liver volumes were similar to early pregnancy, indicative of a return to baseline (Fig. 2*C*). These data provide evidence of postpartum liver involution in women.

While our data showed a statistically significant increase in liver size during pregnancy and a trend toward decrease after weaning, there was heterogeneity in how an individual’s liver size changed with pregnancy and postwean. During pregnancy, we found that 72% (34/47) of women had an average increase in liver volume of ∼20% (Fig. 2*D*, black bars). However, 21% of participants (10/47) had no measurable liver volume change and 6% (3/47) had a reduction in liver volume (Fig. 2*D*, black bars, *SI Appendix*, Table S2). We saw similar heterogeneity with regard to liver volume change from late pregnancy to postwean (Fig. 2*D*, gray bars).

Considering the heterogeneity in liver volume change and what is known about normal rodent liver biology (i.e., liver weight gain with pregnancy and loss postwean) (2), we performed subgroup analyses. We delineated the participants into two groups: “gain-loss,” the observed pattern in the normal rodent, or “not gain-loss” for those that did not display the rodent pattern. 53% of women displayed the anticipated liver “gain-loss” pattern (Fig. 2*E*). The “not gain-loss” group comprised heterogeneous patterns and included three women who lost liver volume during pregnancy and regained postwean, three women with no significant liver volume changes, and one woman each with either continuous liver size loss or gain across the three visits (Fig. 2*F*). Of note, liver volume patterns with pregnancy and postwean did not correlate with a woman’s overall weight gain of pregnancy (*SI Appendix*, Fig. S2).

Upon further exploration, we found that none of the women whose liver changes were similar to the normal rodent pattern of “gain-loss” had gestational hypertension, yet 50% of the “not gain-loss” group did (Fig. 2*F*, dashed lines). Furthermore, measures of insulin sensitivity differed between these groups. Specifically, we found the “gain-loss” participants had greater EGP at late pregnancy (Fig. 2*G*), consistent with published data showing elevated EGP in healthy pregnancy (10). We also found greater glucose disposal rates at late pregnancy in the “gain-loss” group (Fig. 2*H*), consistent with greater insulin sensitivity in the muscle. These data suggest that the “not gain-loss” pattern may be associated with suboptimal gestational metabolic health and gestational hypertension. One question is whether these metabolic parameters impact fetal outcomes. In this cohort, maternal liver size patterns did not correlate with newborn weight, length, or Ponderal index, three common neonatal health measures.

**Fig. 3.**
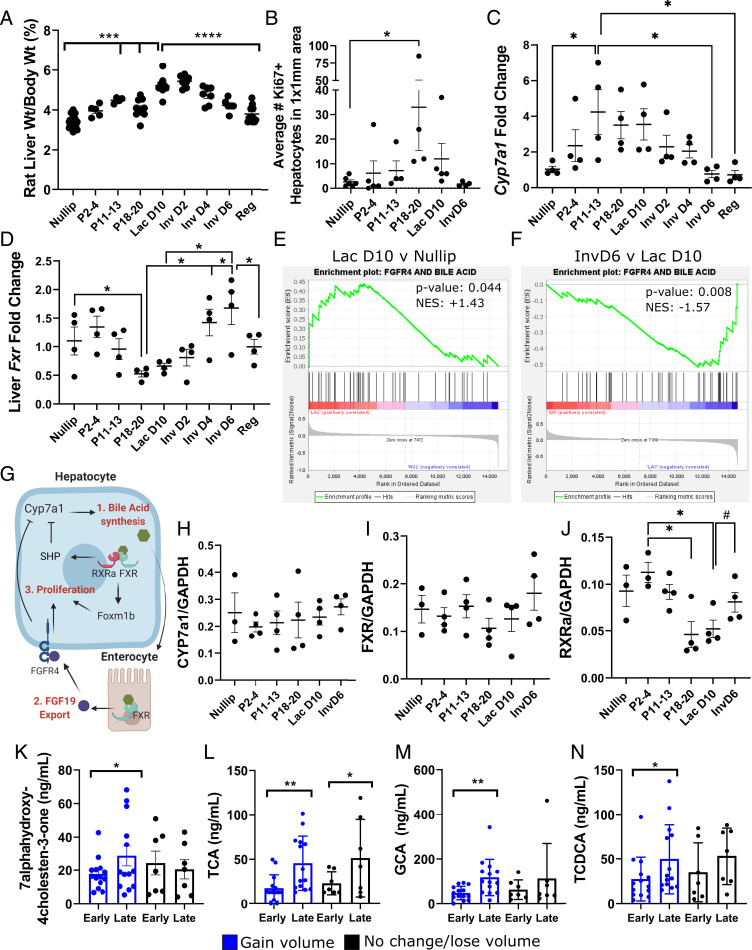
Hepatic bile acid signaling and liver size. (*A*) Rat liver weight normalized to body weight: nulliparous (nullip) *n* = 24; early (P2-4) *n* = 5; middle (P11-13) *n* = 4; and late (P18-20) pregnancy *n* = 10; lactation day 10 (Lac D10) *n* = 9; involution (Inv) D2 *n* = 9; InvD4 *n* = 7; InvD6 *n* = 6; Regressed (Reg) *n* = 14; one-way ANOVA. (*B*) Ki67+ hepatocytes in rat livers, *n* = 3 to 5/group. (*C*) *Cyp7a1* and (*D*) *FXR* mRNA fold change in liver, *n* = 4 per group; one-way ANOVA. Gene set enrichment analysis plots of FGFR4–bile acid gene pathway for (*E*) lactation day 10 versus nulliparous groups and (*F*) involution day 6 versus lactation day 10 groups. (*G*) Model for pregnancy enlargement of liver due to hepatocyte proliferation downstream of bile acid signaling. Protein abundance in whole rat liver of (*H*) CYP7A1, (*I*) FXR, and (*J*) RXRalpha. Data are normalized to GAPDH protein abundance; nullip *n* = 3, P2-4, P11-13, P18-20, Lac D10, and InvD6 *n* = 4/group; *P < 0.05 by one-way ANOVA; # *P* < 0.05 by Student’s *t* test. (*K*) Human 7α-hydroxy-4cholesten-3-one plasma concentrations at early and late pregnancy, separated by liver gain (*n* = 14) and no gain (*n* = 7). Human plasma concentrations of bile acids TCA (*L*), GCA (*M*), and TCDCA (*N*) paired *t* test, *P* value: * < 0.05, ** < 0.01, *** < 0.001, **** < 0.0001.

To investigate the mechanistic relationship between reproductive state and liver size, we utilized a rat model, as previously described (2). We found liver weight increases during pregnancy were greater than expected due to gestational weight gain alone (Fig. 3*A*). These data suggest rat liver weight during pregnancy is unlinked from the “hepatostat,” corroborating our human data (Fig. 1*D*). Next, we confirmed maximum hepatocyte proliferation in the rat livers to occur during pregnancy (Fig. 3*B*), consistent with previous reports (2, 6). Together, these data suggest a physiological model in which increased liver volume of pregnancy is due to increased hepatocyte proliferation that is activated via an unrecognized, pregnancy-mediated developmental program.

As a possible mechanism underlying a pregnancy-associated liver growth program, we investigated bile acid metabolism in rodents. Bile acid signaling contributes to liver regeneration following partial hepatectomy and can control liver size independent of body size (11, 12). Furthermore, bile acids have been shown to regulate hepatocyte proliferation in a pathway dependent on enterocyte-derived fibroblast growth factor 15/19 (11, 13, 14). To investigate if the bile acid pool is modulated by reproductive state, we measured liver *Cyp7a1*, the gene that encodes rate limiting enzyme in bile acid synthesis. We found a three- to fourfold increased expression of *Cyp7a1* with pregnancy, which remained elevated during lactation, followed by a rapid decline with weaning (Fig. 3*C*). Since hepatic FXR signaling acts as a negative regulator of Cyp7a (Fig. 3*D*), we measured hepatic *Fxr*. We found *Fxr* was down-regulated during late pregnancy, when *Cyp7a1* was high, and increased with weaning, when *Cyp7a1* was low (Fig. 3*D*). To further investigate the hypothesis that elevated bile acids contribute to hepatocyte proliferation through activation of FGFR4 signaling, we analyzed an RNA-sequencing dataset from mouse liver at various reproductive stages. We performed gene set enrichment analysis using a custom gene set composed of genes in bile acid metabolism and FGFR4 downstream signaling pathways. Consistent with our hypothesis, we found an enrichment for bile acid–FGFR4 signaling pathways in lactation stage mice compared to nulliparous (Fig. 3*E* and *SI Appendix*, Fig. S3). Additionally, at involution day 6 this bile acid–FGFR4 gene signature was significantly decreased compared to lactation (Fig. 3*F* and *SI Appendix*, Fig. S3). Furthermore, we identified the up-regulation of individual genes involved with bile acid synthesis (*Cyp7a1*, *Cyp8b1*) and proliferation (*Foxm1*) during lactation, which were subsequently down-regulated with involution (*SI Appendix*, Fig. S3). Conversely, genes involved with inhibiting bile acid synthesis (*Rxra*, *Shp*) were reduced during lactation and elevated during involution (*SI Appendix*, Fig. S3). A model depicting a proposed mechanism by which bile acid–FGFR4 signaling increases hepatocyte proliferation is shown (Fig. 3*G*). Because gene expression and protein concentration are not always linked, we evaluated protein abundance for CYP7A1 and FXR. We were unable to validate reproductive-stage regulation of *Cyp7a1* and *FXR* at the protein level (Fig. 3 *H* and *I*). However, based on our mouse RNA-sequencing data that showed regulation of *RXRa* by reproductive stage (*SI Appendix*, Fig. S3), we also measured RXRa protein concentration. RXRa is a known binding partner for FXR that acts as a co-negative regulator of *Cyp7a1* (15, 16). We found that RXRa was significantly reduced at pregnancy days 18 to 20 and lactation day 10, then was increased at involution day 6 (Fig. 3*J*). The decreased gene expression and protein abundance of RXRa might be sufficient to activate *Cyp7A1* gene expression without a corresponding decrease in FXR protein. In sum, these data associate increased bile acids with the physiologic expansion of the liver during pregnancy—consistent with a previous report (12)—and extend these observations to suggest a role for bile acids in regulating liver size during pregnancy, lactation, and weaning.

We then examined associations between liver growth and the bile acid pool in pregnant women by measuring a biomarker of bile acid production and serum bile acid concentrations at early and late pregnancy. Serum concentrations of 7α-hydroxy-4-cholestene-3-one (7αC4), a readout for cholesterol 7α-hydroxylase (Cyp7a1) enzyme activity (17), were significantly increased at late compared to early pregnancy only in the women who had an increase in liver volume during pregnancy (Fig. 3*F*). This finding supports the hypothesis that increased bile acid production during pregnancy may be required for liver size increase. Furthermore, among the women whose liver increased in size during pregnancy, we found increases in several primary bile acids and their conjugates (Fig. 3 *G*–*I*). Of note, changes in secondary bile acids, which are metabolic products of gut bacteria, only weakly correlated with liver volume change (*SI Appendix*, Table S3). In this human cohort, we did not find associations between concentration of plasma FGF19, an enterocyte product shown to induce hepatocyte proliferation and liver growth in rodents (*SI Appendix*, Fig. S4). One potential caveat to our FGF19 analysis is that plasma concentrations of FGF19 may not reflect concentration in the portal vein that links the gut and liver. In sum, these human data are consistent with an increased bile acid pool during pregnancy, which may contribute to the increased liver size observed in pregnancy.

## Discussion

In this study, we find evidence for a previously unreported liver biology in women—namely that during the course of a normal pregnancy cycle liver volume increases during gestation and decreases postwean. Multiple observations and reports demonstrate tight control of liver size in proportion to body size, a phenomenon that has been referred to as the “hepatostat” (4). Yet, in our study, increased liver volume during pregnancy is not accounted for by change in body size. Since liver size is presumed to be directly linked to a physiologic function of the liver (18), our data suggest that a metabolic demand above and beyond body size accounts for increased liver size during pregnancy. Consistent with this hypothesis, in rodents we find that liver size remains elevated through lactation even though body size is reduced compared to late pregnancy.

One potential mechanism controlling liver size during a pregnancy cycle is the circulation of bile acids, which have been shown to modulate liver size independent of the body size hepatostat (19, 20). Such a paradigm where liver size is controlled by bile acid flux would allow for a situation in which body size could become “unlinked” from liver size. The data presented here obtained from rodent models and human correlates support the idea that increased production of primary bile acids during pregnancy and lactation via the Cyp7a1 synthetic pathway leads to hepatocyte proliferation and thus a larger liver. Since the primary function of bile acids is to facilitate fat absorption in the intestine, an increase in liver parenchyma capable of bile acid synthesis would make sense given increased caloric demand during pregnancy and lactation. In sum, our human data are consistent with dynamic size regulation of the liver to accommodate the unique metabolic demands of pregnancy and lactation. Furthermore, our combined human and rodent data suggest a mechanism whereby physiologically regulated bile acid synthesis underlies liver size changes across a pregnancy cycle. Future studies directly testing this mechanism are needed.

Additional factors that could contribute to increased liver size with pregnancy include hormones, such as thyroid hormone, estrogen, and progesterone. Thyroid hormone can induce hepatocyte proliferation (21), yet thyroid hormone does not associate with significant growth of the liver nor is it required for liver regeneration (22). Furthermore, thyroid hormone is known to decrease between early and late pregnancy (23, 24), which is a pattern of expression inconsistent with a role in hepatocyte proliferation. Additionally, previous work has evaluated the impact of estrogen and progesterone on liver size. Administration of pregnancy-relevant concentrations of estrogen and progesterone did not induce liver growth, suggestive that these hormones are not key for increasing liver size during pregnancy (25). Prolactin, which increases through pregnancy and peaks during lactation (26, 27), is known to stimulate hepatocyte proliferation and is associated with accelerated liver regeneration following partial hepatectomy (28–30). A limitation of our study is lack of identification of the molecular mechanism upstream of increased bile acid production during pregnancy and lactation, of which pregnancy hormones could contribute. Intriguingly, there is evidence that prolactin can stimulate bile acid synthesis (31). These prolactin data and our observation that RXRa, a member of the steroid receptor super family, is regulated in a manner consistent with bile acid regulation of liver size, may generate new avenues to pursue.

While the pattern of liver size gain with pregnancy and loss upon weaning was observed in the majority of women in this pregnancy study, we also identified a subset of women for whom liver size did not follow a “gain-loss” pattern. Gestational hypertension and reduced liver insulin sensitivity were more common in this subset. Therefore, an intriguing hypothesis is that a facet of healthy pregnancy is the gain-loss pattern of liver volume. A corollary to this hypothesis is that preexisting and/or pregnancy-specific conditions such as gestational hypertension could underlie the inability of the liver to appropriately respond to pregnancy (32). Of note, gestational hypertension affects 5 to 10% of pregnancies and can progress to preeclampsia with known pathogenic liver involvement in ∼15 to 20% of cases (33). Gestational hypertension is attributed to a vascular disorder that is initiated at the placental interface, specifically due to incomplete maturation of the maternal spiral arteries (34). If related, the question of whether the same pathophysiology that leads to gestational hypertension can also impair the normal liver response to growth cues of pregnancy, or vice versa, remains unknown. On the other hand, albeit a small study, our data show that liver gain with pregnancy is not a requisite for normal fetal growth, as we observed no differences in newborn size between women who did and did not experience liver gain with pregnancy. Future studies would be required to determine if there are any long-term impacts due to a lack of maternal liver growth during gestation, as has been described for other variations in neonatal nutrition and lifetime risk of disease (35–37).

The data presented here show that the human liver responds to a pregnancy cycle in a similar manner to rodents, namely, increased size with pregnancy and lactation, followed by a decrease in size postwean. The process that returns the rodent liver to its prepregnant state, weaning-induced liver involution, promotes breast cancer metastasis to the liver (2, 3). Given that women have an increased risk of liver metastasis if diagnosed with breast cancer within 5 y of pregnancy, we speculate that weaning-induced liver involution creates a prometastatic microenvironment in the liver. Although we cannot definitively demonstrate liver involution in humans, it has recently been demonstrated that the breast undergoes weaning-induced involution in women similar to rodents (38, 39). Therefore, we theorize that there is a conserved mammalian developmental program that links the mammary gland and the liver through a pregnancy cycle, putatively to meet the elevated metabolic demands of pregnancy and lactation. The potential importance of this biology for supporting reproduction and infant health are apparent; however, weaning-induced breast and liver involution may have unanticipated consequences, including the transient increased risk of breast cancer and liver metastasis (2, 40–43). Additional impacts on risk for liver disease may be anticipated given known disparities in liver disease by sex, including increased risk for acute liver failure and autoimmune liver conditions in women (1).

A key strength of our study is that each woman serves as her own control, allowing us to see how an individual’s liver changes during a pregnancy cycle. However, our human cohort study cannot draw mechanistic conclusions because it was purely observational. Additionally, these data were generated in a small, predominately White, non-Hispanic cohort and require validation in a larger study with a diverse population to generalize these findings.

In summary, this work describes an observation in normal women, specifically increased liver size with pregnancy and decreased size postwean, putatively to accommodate the dramatic changes in metabolic demands across a pregnancy–lactation–wean cycle. These findings demonstrate reproductive control of liver size and function in women and concur with recent observations in rodents, suggesting a conserved liver biology. The question of whether this described liver biology has implications for maternal health during pregnancy or sex-specific risk for liver disease remains to be determined (1, 44, 45). However, our evidence suggest weaning-induced liver involution in women, which if validated, may lead to improved understanding of the high rates of liver metastasis observed in young postpartum breast cancer patients (2).

## Materials and Methods

### Recruitment.

#### Prospective cohort.

We conducted a prospective cohort study of pregnant women receiving care at Kaiser Permanente Northwest (KPNW) or Oregon Health & Science University (OHSU). All study activities were approved by Institutional Review Boards at KPNW (no. 3993) and OHSU (no. 10438 and 15264). Recruitment started in December 2014 and was completed in August 2017. KPNW members who met the study inclusion criteria were identified weekly using the electronic health record (EHR). Eligible participants were mailed a recruitment letter and received a follow-up phone call a week later. During this telephone call, study personnel conducted additional eligibility screening and scheduled an explanatory visit. If patients consented at the explanatory visit, this was followed by two visits between 12 to 16 wk of gestation and two visits between 32 to 36 wk of gestation. Participants who completed all pregnancy study visits had the option to complete a postwean study visit, between 3–12 mo after weaning.

#### Inclusion/exclusion criteria.

Patients were eligible for the study if they were between 18 to 45 y of age, were less than 12 wk pregnant with a singleton gestation at time of enrollment, had a body mass index (BMI) between 18.5 kg/m^2^ and 38 kg/m^2^, and were fluent English speakers. Participants were excluded if they had any of the following conditions or symptoms: contraindications to MRI study (e.g., claustrophobia, metal implants); pregestational diabetes; gestational diabetes; history of bariatric surgery or other medical conditions requiring specialized nutritional care; anemia; current history of drug, tobacco, or alcohol use; maternal rheumatologic or chronic inflammatory state; or chronic hypertension.

### Measures.

Data for this paper were collected at three study visits: one at 12 to 16 wk of gestation, one at 32 to 37 wk of gestation, and one between 3–12 mo postwean. Height was measured at the first visit to allow for calculation of BMI; weight was measured using a calibrated scale at each visit. Demographic variables, including parity and preconception BMI, were extracted from the EHR.

### Air Displacement Plethysmography.

Air displacement plethysmography (BOD POD, COSMED USA, Inc.) was used to determine participants’ fat mass, fat-free mass, and percent body fat at each visit. Participants first changed into a bathing suit or spandex clothing and a swimming cap. They then sat inside the BOD POD while the air displaced by the body was measured. Results included total mass and body density. Fat mass and fat-free mass were estimated using van Raaj’s pregnancy equations to account for changes in the density of fat-free mass during pregnancy (47, 48).

### MRI Acquisition.

MRI and spectroscopy data were collected using a Siemens Prisma Fit 3T whole-body system (Siemens Healthineers) at the Advanced Imaging Research Center at OHSU. Abdominal MR data were acquired in two stations, the first centered at umbilicus and the second centered over the xyphoid process, to acquire MRI and liver magnetic resonance spectroscopy (MRS). Siemens flexible 18-channel array and spine array receiver coils with body-coil transmission were used. The abdominal MRI protocol included a T1-weighted gradient-echo sequence (TE = 2.5 ms, TR = 140 ms, flip-angle = 90°, (1.25 mm)^2^ in plane resolution, 30 slices with 6 mm thickness) acquired in two-breath holds of ∼18 s each. The liver T1-weighted MRI protocol was acquired with identical parameters to the abdominal T1 volume but with a variable number of slices to cover the entire extent of the liver.

### MRI Processing.

The T1-weighted MRI data sets of abdomen and liver were manually spliced together with affine transformations and overlapping slice elimination. The top of the liver and the L-4/5 intervertebral disk were identified as the upper and lower bounds, respectively, for the segmentation analysis for abdominal visceral and subcutaneous fat volumes.

Abdominal T1-w MRI volumes were segmented into five classes: unlabeled, subcutaneous adipose tissue (SAT), VAT, muscle, and organ (including all other abdominal volume). A custom Python pipeline was used to create an initial automated segmentation using inputs from the umbilicus T1-weighted volumes, the liver T1-weighted volumes, and an 11-slice manual segmentation label map, the merged T1-weighted MRI data set, and the affine transforms that map individual volume acquisitions to the merged image space. Manually generated uterus/placenta and liver masks were created as these two regions have high rates of false positives for classification as adipose tissue.

Processing within the pipeline made use of the following Python libraries: Nipype (49), the Advanced Normalization Tools (50), the Insight Toolkit (51), Scikit-image (52), Scikit-learn (53), and SciPy (54). Following N4 bias field correction, steps in the segmentation pipeline relied upon intensity thresholding and morphological operations. The muscle mask was generated with a compact watershed algorithm seeded with the muscle mask from the 11-slice segmentation. SAT masking made use of the geodesic active contours algorithm (55), coupled with dilation and erosion steps to distinguish the SAT from internal VAT. VAT was taken as the difference between the total adipose mask and the SAT mask. Segmentation masks output from the automated pipeline subsequently underwent slice-by-slice manual review followed by manual refinement by a single analyst ( J.Q.P.) using the 3D Slicer software package to ensure accuracy of VAT and SAT masks placements.

Liver segmentation was manually conducted separately using the OsiriX and Image J software programs.

### Liver Volume Determination.

Image analysis was performed using OsiriX (OsiriX Imaging Software) software and Image J software (NIH). For volume estimation, 3D-VIBE (a T1-weighted FLASH technique with fat selective prepulse) sequences were used. The liver was identified on each image, and the outline of the liver tissue annotated by freehand region of interest estimation by operators trained by a body radiologist with over 10 y of experience in MRI of the liver. This allowed for the generation of a liver area on each slice. Liver volume was calculated by multiplying the estimated area of each slice by the interval between slices, summing all volumes containing liver for the total liver volume (46).

Liver volume determinations were performed by two blinded operators. Operators independently measured liver volumes for five cases with two MRI scans per case (early and late pregnancy). The observed interoperator variability (*SI Appendix*, Table S2) was used to benchmark values that are within the range of measurement error, in this case +7 to −7%.

### MRS.

IHL was measured using 1H single-voxel MRS, following MRI. Liver MRS voxels were positioned within the right lobe with voxel sizes ranging from 18 to 24 cm^3^.

Liver spectra were collected using a point-resolved spectroscopy single-voxel spectroscopy sequence (TR = 5 s, TE = 30 ms, 1,024 points, 2,000 Hz spectral width). The long repetition time ensured fully relaxed water signal (99.2%), because it serves as an internal standard for quantification. Three separately acquired MRS series were run, each during a 10-s breath hold.

MRS analysis was conducted using the advanced method for accurate, robust, and efficient spectral time-domain fitting module within the jMRUI software program. All spectral fits were inspected and rerun with additional constraints if fitting contained errors. IHL is expressed as a proportion of primary lipid peak to water peak areas.

### Hyperinsulinemic-Euglycemic Clamp.

Hyperinsulinemic-euglycemic clamp with coinfusion of [6,6-2H2] glucose was used to determine whole-body and skeletal muscle insulin sensitivity (Rd) and EGP (56, 57). Subjects were advised regarding a standard diet consisting of 30% of total calories from fat sources, 15% from protein, and 55% from carbohydrates for the 3 d before study. Following an 11-h overnight fast, subjects were admitted to the OHSU Clinical and Translational Research Center where a hyperinsulinemic-euglycemic clamp was performed. At 0600, an intravenous catheter was placed in one arm for infusions and in the contralateral hand for blood withdrawal and warmed to 70 °C using a warming mitt for sampling of arterialized venous blood. A primed constant infusion of [6,6-2H2] glucose (Cambridge Isotope Laboratories) was infused at 0.133 mL/min and an enrichment intended to achieve ∼1.0 mol percent excess for all subjects. The basal infusion of [6,6-2H2] glucose was continued for 2 h, and plasma samples were obtained from 90 to 120 min to estimate basal EGP and fasting insulin concentration. Basal EGP was calculated according to the steady-state equations of Steele (58). At the completion of the 2-h infusion glucose isotope, a primed, constant infusion of regular insulin at 40 mU/m2/min was started. Plasma glucose was maintained at 90 mg/dL for the remaining 2 h. During the final 30 min of the clamp, blood samples were obtained every 5 min for isotope analysis. Suppression of EGP by insulin infusion during the 2-h clamp was estimated using the method developed by Black (59). EGP, Rd, and M value were adjusted for insulin level (mU/L) and fat-free mass (kg).

#### Labs.

Venipuncture was used to obtain blood samples with participants in the fasting state. The following measures were assessed and run in the Laboratory Core of the Oregon Clinical and Translation Research Institute: comprehensive metabolic panel, lipid panel, free fatty acids, liver function tests, glucose, and insulin. Insulin was assessed by radioimmunoassay (Mercodia AB) and glucose by a Hexokinase based colorimetric assay (Stanbio laboratory).

### Bile Acid Profiling.

Bile acid profiling was performed in the OHSU Bioanalytical Shared Resource/Pharmacokinetics Core. Plasma samples from early and late pregnancy were utilized to quantify plasma bile acids and 7α-hydroxy-4-cholesten-3-one using liquid chromatography–tandem mass spectrometry (LC-MS/MS). Quantification of plasma bile acids was performed with a 4000 QTRAP hybrid triple quadrupole-linear ion trap mass spectrometer (SCIEX) operating with electrospray ionization (ESI) in the negative mode. The mass spectrometer was interfaced to a Shimadzu high-performance liquid chromatography (HPLC) system consisting of SIL-20AC XR auto-sampler and LC-20AD XR LC pumps. Analyte separation was achieved using a gradient HPLC method and Luna 2.5u C18 (2)-HST 50 × 2 mm column (Phenomenex) kept at 50 °C with a Shimadzu CTO-20AC column oven.

The stable isotope dilution LC-MS/MS method to quantify plasma bile acids was previously described (60). In brief, plasma was spiked with internal standards, and bile acids were measured following protein precipitation and extraction with methanol, centrifugation, and filtration of the supernatant. Calibrants were prepared in charcoal stripped matrix (SP1070 from Golden West Biological) using authentic bile acid and conjugate standards (obtained from Toronto Research Chemicals and Cerilliant).

Data were acquired using SCIEX Analyst 1.6.2 and analyzed using SCIEX MultiQuant 3.0.3 software. Sample values were calculated from calibration curves generated from the peak area ratio of the analyte to internal standard versus analyte concentration that was fit to a linear equation with 1/× weighting. The following bile acids were measured: Taurocholic acid (TCA), Glycocholic acid (GCA), Taurochenodeoxycholic acid (TCDCA), Glycochenodeoxycholic acid (GCDCA), Ursodeoxycholic acid (UDCA), Cholic acid (CA), Chenodeoxycholic acid (CDCA), Deoxycholic acid (DCA), and Lithocholic acid (LCA). Compounds were quantified with multiple reaction monitoring and transitions optimized by infusion of pure compounds.

Plasma 7α-hydroxy-4-cholesten-3-one was determined by LC-MS/MS following protein precipitation and extraction with acetonitrile. To each 100 µL sample of EDTA plasma was added 1 ng of internal standard 7α-hydroxy-4-cholesten-3-one-d7 (prepared at 0.2 ng/µL in methanol) and 300 µL of acetonitrile. The samples were vortex mixed and centrifuged at 12,000 × *g* for 10 min. The supernatant was removed and filtered prior to injection for analysis with LC-MS/MS.

Calibration standards were prepared across the range 1 to 100 ng/mL in charcoal stripped plasma SP1070 (Golden West Biological) using authentic 7α-hydroxy-4-cholesten-3-one (obtained from Toronto Research Chemicals).

LC-MS/MS was performed using a 5500 QTRAP hybrid triple quadrupole-linear ion trap mass spectrometer (SCIEX) with ESI in the positive mode. The mass spectrometer was interfaced to a Shimadzu HPLC system consisting of SIL-20AC XR auto-sampler and LC-20AD XR LC pumps. The 5500 QTRAP was operated with the following settings: source voltage 4500 kV, GS1 40, GS2 30, CUR 40, TEM 650, and CAD gas high.

Analyte separation was achieved using a Gemini 3u C6-Phenyl 110A 100 × 2 mm column (Phenomenex) kept at 35 °C using a Shimadzu CTO-20AC column oven. The gradient mobile phase was delivered at a flow rate of 0.4 mL/min and consisted of two solvents: A, 0.1% formic acid in water; B: 0.1% formic acid in acetonitrile. The initial concentration of solvent B was 40% followed by a linear increase to 95% B in 10 min, this was held for 2 min, then decreased back to 40% B over 0.1 min, then held for 3 min. The retention time for 7α-hydroxy-4-cholesten-3-one was 8.2 min.

Data were acquired using SCIEX Analyst 1.6.2 and analyzed using SCIEX MultiQuant 3.0.3 software. Sample values were calculated from calibration curves generated from the peak area ratio of the analyte to internal standard versus analyte concentration that was fit to a linear equation with 1/× weighting.

### FGF19 ELISA.

Human serum concentration of FGF-19 was determined using the Human FGF-19 Quantikine enzyme-linked immunoassay (ELISA) (R&D Systems, DF1900). Assay was completed according to manufacturer’s instructions with samples run in duplicate.

### Rodent Studies.

#### Postpartum rodent model.

The University of Colorado Anschutz Medical Campus approved rat procedures. Age-matched Sprague Dawley female rats (Harlan) were housed and bred as described (61). For tissue collection, rats were euthanized across groups by CO_2_ asphyxiation and cardiac puncture. Whole livers were removed, washed 3× in 1× phosphate-buffered saline (PBS), and tissues weighed. Left lobes were fixed in 10% neutral buffered formalin (Anatech ltd) and processed for formalin fixed, paraffin embedded, and caudate lobes were flash frozen on liquid nitrogen for protein and RNA extraction. Oregon Health & Science University Institutional Animal Care and Use Committees approved mouse procudures. Age-matched Balb/c female mice (Charles River Laboratories, The Jackson Laboratory) were housed and bred as described (61). For tissue collection, mice were euthanized across groups by CO_2_ asphyxiation and cardiac puncture. Whole livers were removed, washed 3× in 1× PBS and tissues weighed. Caudate lobes were flash frozen on liquid nitrogen for RNA extraction.

#### Immunohistochemistry.

Immunohistochemical detection was performed as described (62). Briefly, tissues were deparaffinized, rehydrated, and heat-mediated antigen retrieval was performed with EDTA for 5 min at 125 °C. Primary antibodies used were as follows: Ki67 (Neomarkers RM-9106-s, 1:50) for 2 h at room temperature (RT) and Adipophilin (LS-B2168/34250 Lifespan Biosciences, 1:400) for 1 h at RT. Secondary antibody was anti-rabbit (Agilent Envision+ K4003, RTU), used for Ki67 at 1 h at RT and for Adipophilin at 30 min at RT. DAB chromogen (Agilent, K346889-2) with hematoxylin counter stain (Agilent, S330130-2) was used to visualize positive stain. Stained sections were scanned using the Aperio AT2 slide scanner (Leica Biosystems). Number of Ki67+ hepatocytes were counted in five 1 × 1 mm areas. Adipophilin signal quantification was performed by Aperio ImageScope version 12.1.0.5029 as described previously (63). All analyses were done by investigators blinded to group.

#### Real-time qRT-PCR.

RNA was isolated from flash frozen rat liver for complementary DNA (cDNA) synthesis and qPCR. One microgram total RNA was used for RT-mediated synthesis of cDNA using SuperScript II RT (Invitrogen) and random hexamer primers for Cyp7a and SuperScript IV (Invitrogen) for FXR. qPCR for rat Cyp7a and reference gene GAPDH was performed using FastStart Essential DNA Green Master (Roche) in an Applied Biosystems theromocycler with 45 cycles of 95 °C for 20 s, 60 °C for 40 s, and 72 °C for 20 s. Rat primer sequences were as follows: Cyp7a, forward CTGTCATACCACAAAGTCTTATGTCA and reverse ATGCTTCTGTGTCCAAATGCC; GAPDH forward CGCTGGTGCTGAGTATGTCG and reverse CTGTGGTCATGAGCCCTTCC.

qPCR for rat FXR and reference gene GAPDH was performed using SsoAdvanced Unviersal SYBR Green Supermix (BioRad) in the ViiA 7 Real-Team PCR System (Thermo Fisher) with the following times: 95 °C for 2 min, 40 cycles of 95 °C for 15 s and 56 °C for 60 s, then 95 °C for 15 s, 61 °C for 60 s, and 95 °C for 15 s. Rat primer sequences were as follows: FXR, forward AGGCCATGTTCCTTCGTTCA and reverse TTCAGCTCCCCGACACTTTT; GAPDH, forward ACCACAGTCCATGCCATCAC and reverse TCCACCACCCTGTTGCTGTA.

#### Immunoblotting.

Rat liver protein lysates in radioimmunoprecipitation assay buffer were separated by Wes automated gel electrophoresis system (Protein Simple). Primary antibodies and dilutions were as follows: CYP7A1 (Abcam no. ab234982, 1:20), FXR (Thermo Fisher Invitrogen no. 417200, 1:50), RXRa (Abcam no. ab125001, 1:20), and GAPDH (Cell Signaling Technology no. 2118, 1:20). Protein input for CYP7A1 and FXR assays was 0.25 mg/mL. Protein input for RXR assay was 0.5 mg/mL. GAPDH assays used both 0.25 mg/mL and 0.5 mg/mL protein input. Anti-rabbit (Protein Simple no. 042-206, RTU) or anti-mouse (Protein Simple no. 042-205, RTU) horseradish peroxidase–conjugated secondary antibodies were utilized, followed by chemiluminescent substrate (Protein Simple no. PS-CS01, Luminol-S, Peroxide). Signal was detected using the Wes System camera. Immunoblot electrophoretograms were analyzed by Compass Software (Protein Simple).

#### RNA-sequencing.

RNA was isolated from flash frozen whole murine liver using the Direct-zol RNA MiniPrep kit (Zymo Research no. R2051). An input of 100 ng RNA was used for library preparation. Library construction was performed by Novogene using a NEBNext Ultra RNA Library Prep Kit for Illumina (cat no. E7420S, New England Biolabs) according to the manufacturer’s protocol. Briefly, messenger RNA (mRNA) was enriched using oligo(dT) beads followed by two rounds of purification and fragmented randomly by adding fragmentation buffer. The first-strand cDNA was synthesized using random hexamers primer, after which a custom second-strand synthesis buffer (Illumina), dNTPs, RNase H, and DNA polymerase I were added to generate the second-strand (double stranded cDNA). After a series of terminal repair, poly-adenylation, and sequencing adaptor ligation, the double-stranded cDNA library was completed following size selection and PCR enrichment. The resulting 250- to 350-base pair (bp) insert libraries were quantified using a Qubit 2.0 fluorometer (Thermo Fisher Scientific) and qPCR. Size distribution was analyzed using an Agilent 2100 Bioanalyzer (Agilent Technologies). Qualified libraries were sequenced on an Illumina Novaseq6000 Platform (Illumina) using a paired-end 150 run (2 × 150 bases). The raw fastq files were first quality checked using FastQC (version 0.11.8) software. Fastq files were aligned to mm10 mouse reference genome (GRCm38.39) and per-gene counts quantified by RNA-Seq by Expectation-Maximization (RSEM) (version 1.3.1) based on the gene annotation Mus_musculus.GRCm38.89.chr.gtf. Differential gene expression analysis was performed using DESeq2 (version 1.22.2) (64). Gene expression differences were considered significant if passing the following criteria: adjusted *P* value < 0.05, log2(fold change) ≥ 1. Custom gene set for GSEA analysis was built from curated gene lists available from Molecular Signature Database (http://www.gsea-msigdb.org/gsea/index.jsp). Specifically, the gene set was composed from the following: REACTOME_DOWNSTREAM_SIGNALING_OF_ACTIVATED_FGFR4 and REACTOME_BILE_ACID_AND_BILE_SALT_METABOLISM. Gene Set Enrichment Analysis (GSEA) analysis was performed with GSEA software developed by the University of California San Diego and Broad Institute (65, 66).

## Supplementary Material

Supplementary File

## Data Availability

Raw RNA-sequencing data of mouse liver tissues performed in this study have been deposited in the Gene Expression Omnibus database under accession code GSE188680. All other study data are included in the article and/or *SI Appendix*.
