# Supplementary material for: Pregnancy and weaning regulate human maternal liver size and function
Source: Proc Natl Acad Sci U S A. 2021 Nov 22;118(48):e2107269118. doi: 10.1073/pnas.2107269118 (PMC8640831; doi:10.1073/pnas.2107269118)
Supplement: Supplementary File [file pnas.2107269118.sapp.pdf]

SFig 1

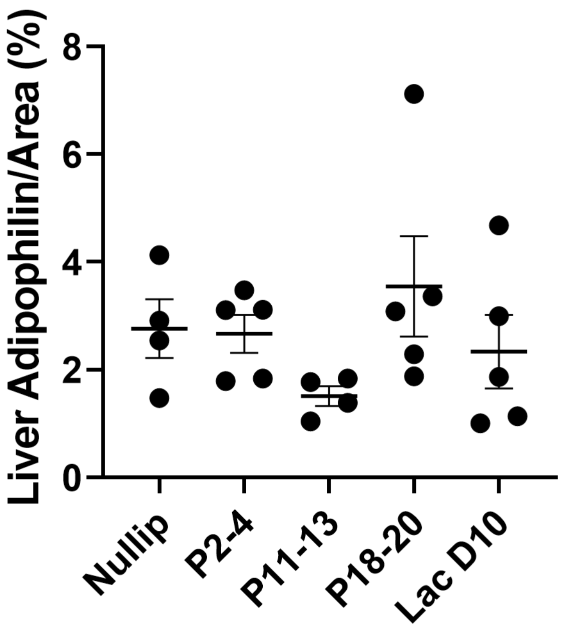

SFig 1 Quantification of adipophilin IHC staining in rat livers, n=4-5/group.

SFig 2

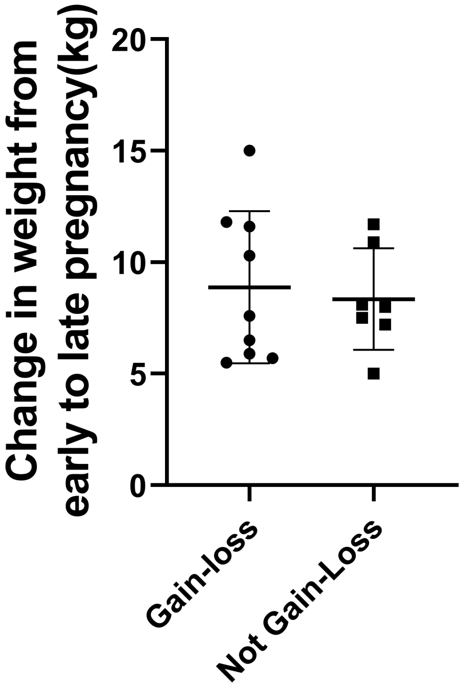

SFig 2 Change in weight from early to late pregnancy in participants who completed all 3 study visits, separated by whether their liver volume followed the gain-loss or not gain-loss pattern during pregnancy and post-wean.

SFig 3

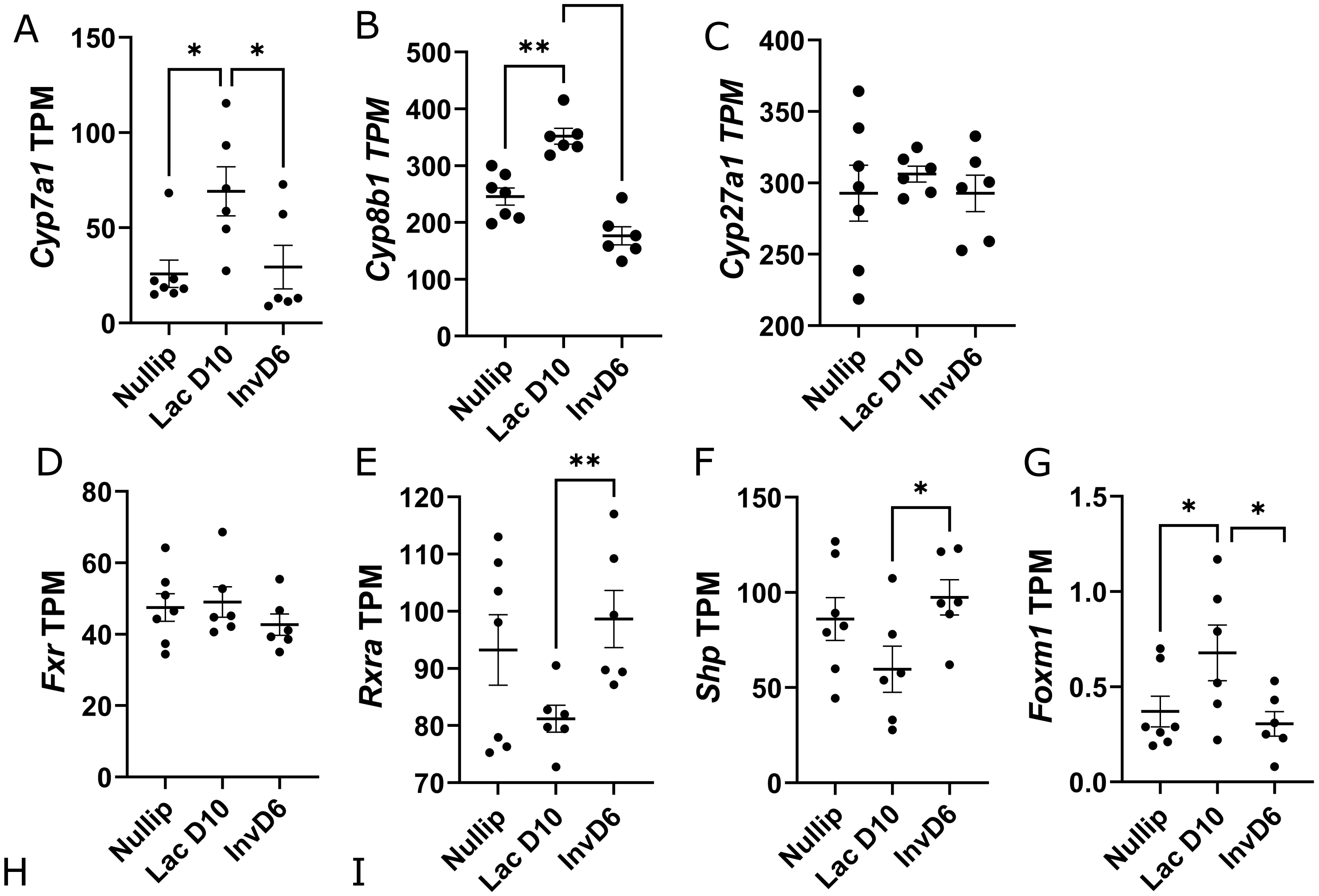

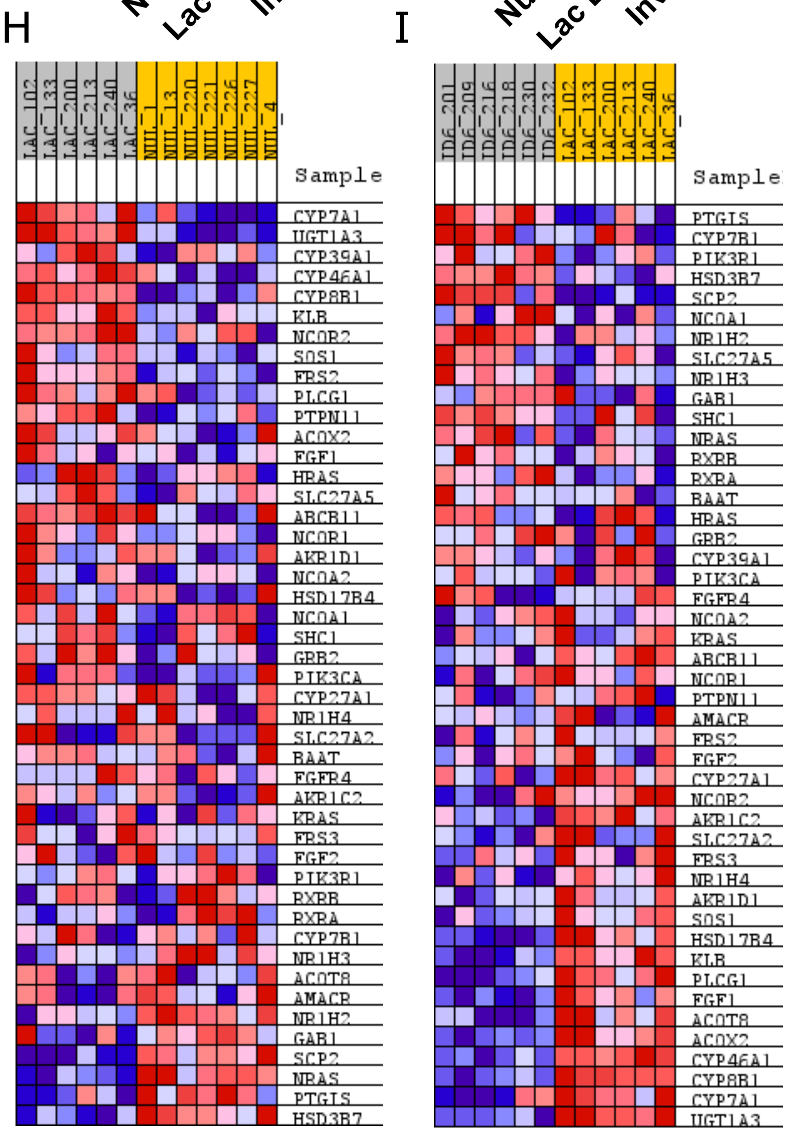

SFig 3 RNA-sequencing analysis of bile acid signaling in the mouse liver. Transcripts per million reads for (A) Cyp7a1, (B) Cyp8b1, (C) Cyp27a1, (D) Fxr, (E) Rxra, (F) Shp, and (G) Foxm1 in nulliparous n=7, lac D10 n=6, and InvD6 n=6. Unpaired T-test, P value: * < 0.05, ** <0.01, **** < 0.0001. Heatmap plots from gene set enrichment analyses of FGFR4-bile acid gene pathway for (H) lactation day 10 versus nulliparous groups and (I) involution day 6 versus lactation day 10 groups.

SFig 4

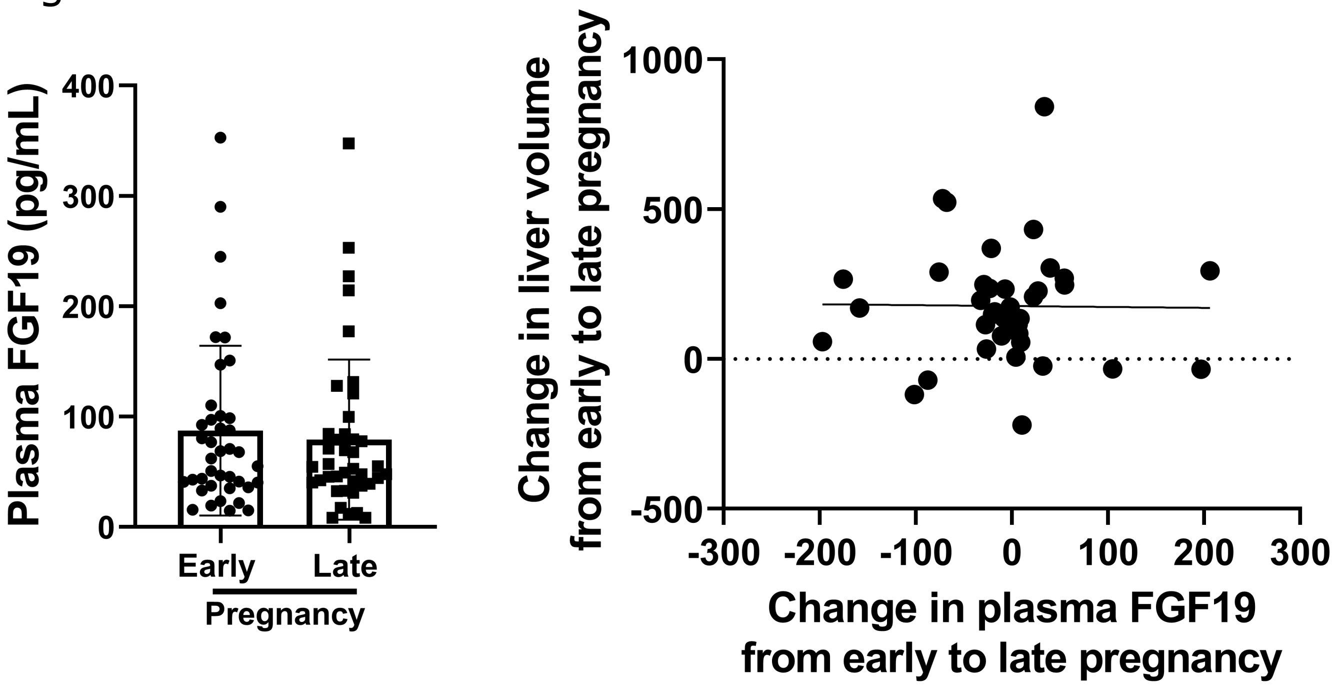

SFig 4 FGF19 Quantification. (A) Quantification of plasma FGF19 by ELISA at early and late pregnancy time point. (B)Pearson's correlation of change in FGF19 and change in liver volume.

Supplemental Table 1

	Full Liver Volume Population (N=47) N	Liver Volume measured at pregnancy visits *only* (N=30) N	Liver volume measured at pregnancy and postpartum visits, (N=17) N
**Age (years)**			
mean (SD) *[min-max]*	30.2 (4.6) [19.0-39.0]	29.6 (4.7) [19.0-39.0]	31.2 (4.4) [22.0-38.0]
**Preconception BMI (kg/mg^2)**			
mean (SD) *[min-max]*	25.9 (4.4) [18.4-35.9]	26.4 (4.2) [18.4-35.6]	25.2 (4.7) [20.0-35.9]
Normal or Overweight (<30)	40 (85.1)	26 (86.7)	14 (82.4)
Obese (≥30)	7 (14.9)	4 (13.3)	3 (17.6)
**BMI at late pregnancy (kg/m^2)**			
mean (SD) [min-max]	29.5 (4.1) [22.9-38.9]	30.4 (3.7) [22.9-38.9]	28.0 (4.3) [22.9-37.1]
Normal or Overweight (<30)	27 (57.4)	15 (50.0)	12 (70.6)
Obese (≥30)	20 (42.6)	15 (50.0)	5 (29.4)
**Parity, N (%)**			
0	28 (59.6)	21 (70.0)	7 (41.2)
1	10 (21.3)	3 (10.0)	7 (41.2)
>1	9 (19.1)	6 (20.0)	3 (17.6)
**Race, N (%)**			
White only	40 (85.1)	25 (83.3)	15 (88.2)
Multiple races	3 (6.4)	1 (3.3)	2 (11.8)
Unknown	4 (8.5)	4 (13.3)	
**Ethnicity, N (%)**			
Hispanic	6 (12.8)	5 (16.7)	1 (5.9)
Non-Hispanic	41 (87.2)	25 (83.3)	16 (94.1)
**Gestational Age at Early Pregnancy (weeks)**			
mean (SD) *[min-max]*	15.6 (0.8) [12.9-17.6]	15.4 (0.8) [12.9-16.6]	16.1 (0.8) [14.7-17.6]
**Gestational Age at Late Pregnancy (weeks)**			
mean (SD) *[min-max]*	34.3 (1.5) [31.6-37.7]	34.5 (1.5) [32.3-37.7]	33.8 (1.3) [31.6-36.1]
**Gestational hypertension/pre-eclampsia**			
Yes, N (%)	6 (12.8)	2 (6.7)	4 (23.5)
No, N (%)	41 (87.2)	28 (93.3)	13 (76.5)
**Intrahepatic cholestasis of pregnancy**			
Yes, N (%)	2 (4.3)	2 (6.7)	0
No, N (%)	45 (95.7)	28 (93.3)	17 (100.0)
**Newborn weight (kg)**			
mean (SD) *[min-max]*	3.4 (0.5) [2.0-4.5]	3.4 (0.4) [2.6-4.5]	3.5 (0.7) [2.0-4.5]
**Newborn length (cm)**			
mean (SD) *[min-max]*	51.1 (2.8) [44.0-56.0]	51.3 (2.4) [47.0-56.0]	50.9 (3.4) [44.0-56.0]
**Ponderal index (kg/m^3)**			
mean (SD) *[min-max]*	25.5 (2.7) [20.4-32.1]	25.2 (2.9) [20.4-32.1]	26.0 (2.5) [21.9-29.6]

Supplemental Table 2

Interoperator Variability in Liver Volume Measurement				
**Case**	**Output**	**Operator #1**	**Operator #2**	**Difference between operator #1 and #2**
**100283**	Early Pregnancy (cm^3)	1142	1143	1
	Late Pregnancy (cm^3)	1376	1341	-35
	Late-Early (cm^3)	234	198	-36
	% Change	20.5	17.3	**-3.2**
**100445**	Early Pregnancy (cm^3)	948	986	38
	Late Pregnancy (cm^3)	982	999	17
	Late-Early (cm^3)	34	13	-21
	% Change	3.6	1.3	**-2.2**
**100515**	Early Pregnancy (cm^3)	1203	1268	65
	Late Pregnancy (cm^3)	1727	1709	-18
	Late-Early (cm^3)	524	441	-83
	% Change	43.6	34.8	**-8.7**
**100662**	Early Pregnancy (cm^3)	1015	1026	11
	Late Pregnancy (cm^3)	971	966	-5
	Late-Early (cm^3)	-44	-60	-16
	% Change	-4.33	-5.89	**-1.55**
**101089**	Early Pregnancy (cm^3)	1300	1322	22
	Late Pregnancy (cm^3)	1835	1816	-19
	Late-Early (cm^3)	535	494	-41
	% Change	41.2	37.3	**-3.8**
		**Average Difference in % Change**		**-3.9**
		**Standard Deviation in % Change**		**2.8**

Supplemental Table 3

	TCA	GCA	CA	TCDCA	GCDCA	CDCA	*UDCA*	*DCA*	*LCA*	Total Serum Bile Acids	Total Primary Bile Acids	Total Secondary Bile Acids
**Early vs. Late**	P<0.001	P<0.01	n.s.	P<0.05	n.s.	n.s.	n.s.	n.s.	n.s.	n.s.	n.s.	n.s.
**Only Participants with Increase in Volume**	P<0.01	P<0.01	n.s.	P<0.05	n.s.	n.s.	n.s.	n.s.	p<0.05	Trending (p=0.0551)	Trending (p=0.0574)	n.s.
**Only Participants with No Change or Loss in Volume**	P<0.05	n.s.	n.s.	n.s	n.s.	n.s.	n.s.	n.s.	n.s.	n.s.	n.s.	n.s.
